# “Thanks to my activists Friends”: a qualitative study of perspectives of young adults and professionals on the factors related to seeking support among victims of sexual violence in Spain

**DOI:** 10.1186/s13690-024-01319-z

**Published:** 2024-06-21

**Authors:** Eva Durán-Martín, Belén Sanz-Barbero, Aitana Muñoz-Haba, Sebastià March, Carmen Vives-Cases

**Affiliations:** 1 International Doctoral School of the Universidad Nacional de Educación a Distancia and Instituto Mixto de la Escuela Nacional de Salud (UNED- IMIENS), Madrid, 28015 Spain; 2 National School of Public Health, Instituto de Salud Carlos III, Madrid, 28029 Spain; 3 CIBER of Epidemiology and Public Health (CIBERESP), Madrid, 28029 Spain; 4 Cooperativa APLICA, Madrid, 28015 Spain; 5 Department of Community Nursing, Preventive Medicine and Public Health an History of Science, Universidad de Alicante (UA), Alicante, 03690 Spain

**Keywords:** Sexual violence, Young adult, Health assets, Social and health resources

## Abstract

**Background:**

Sexual violence (SV) is a public health problem with high prevalence among the young population. The use of formal resources for SV care (e.g. institutional services) is low in this age group. This study applies a framework of health assets to identify the factors that positively influence the search for support for SV among young people, the functions of that support and the level of proximity as articulated by both young people and professionals.

**Methods:**

A qualitative study was conducted through 38 semi-structured interviews with young people and professionals from SV resource centers and/or care services for the young population in Spain. A thematic theoretical analysis was conducted, embedded in inductive insights emerging from the data, following a particular adaptation of the constant comparative method, under the grounded theory approach. This analysis was carried out by coding the interview transcripts with the support of Atlas.ti.

**Results:**

Young women identified assets, categorized as feminist, that they believe they are essential in the search for formal support services for SV. However, unlike young men, they considered the police and judicial system to be barriers and not assets. There were also differences between the young people and professionals in identifying assets. Young people also considered their partner and traditional media as health assets, in contrast to professionals who did not identify these as such valuable resources in the help-seeking process. Informal assets, such as family, friends and the internet are considered close resources. In contrast, specialized care services for gender-based violence/SV and the judicial and police systems were perceived as more distant resources among the young interviewees.

**Conclusions:**

This study shows similarities and discrepancies among young women and men and SV response professionals in identifying assets for seeking SV support among youth. The study shows an inverse relationship between perceptions of proximity and the level of formality of the asset. This study also contributes to map the relationships and information exchanges between assets. It is advisable to inform professionals about the assets that professionals do not acknowledge, and about actions that allow young people to access formal resources.

**Supplementary Information:**

The online version contains supplementary material available at 10.1186/s13690-024-01319-z.

**Table:** 

Text box 1. Contributions to the literature
• Access to sexual violence resources among young people has not been previously studied from the health assets model. This model brings added value to understanding the help-seeking process and improving access because young people themselves identify themselves as assets and facilitators for victims of sexual violence at different stages of the violence process
• Young people identify the partner as a factor that enhances the ability to seek help in a situation of SV outside the relationship
• Professionals and young men do not identify essential assets for young women (feminist assets). In practice, this implies increasing training and awareness-raising on SV
• It has been seen that there is a gap between institutions and feminist organisations, but also how young people reproduce gender roles when they identify themselves as active and supportive in a situation of SV

## Background

Sexual violence (SV) is a highly prevalent public health problem around the world [1]. SV is defined as any act or attempt to consummate a forced or coerced sexual act without express consent, unwanted sexual comments, or insinuations or actions to market or use a person’s sexuality by means of coercion (psychological pressure, blackmail) [2–4]. SV occurs in different settings, including in the digital environment. It can happen outside of an established relationship (non-partner SV) and within it (SV-IPV) [2–4]. In the European Union, 11% of women reported having been exposed to SV at some point in their lives [5]. In Spain, lifetime SV prevalence is around 13.7% [6]. Young women (16–24 years of age) have experienced a higher prevalence of SV in their lifetime than adult women (25 years of age or older). This is repeated in the context of the partner (12.4% vs. 8.9%) as well as outside the relationship (11% vs. 6%) [6].

Young men and women exposed to SV tend to seek support from family and friends more than from formal support networks (e.g. institutional services) [7–10]. The lack of trust towards formal support resources, the naturalization of SV in relationships, lack of knowledge about such resources and feeling a lack of familiarity with support professionals have been previously identified as barriers to using SV-related formal support resources [10–15]. From a health promotion perspective, Morgan and Zigilo suggest that an exclusive focus on this “deficit” model, which analyzes barriers, impedes the inclusion of other health assets (HA) that can play a role in addressing the response to sexual violence from other perspectives [16]. They have proposed complementing this approach with a health asset (HA) approach, based on identifying and addressing actions from a more positive perspective.

Health assets are defined as “*any factor (or resource) which enhances the ability of individuals, groups, communities, populations, social systems and/or institutions to maintain and sustain health and well-being and to help to reduce health inequities. These assets can operate at the level of the individual, group, community and/or population as protective (or promoting) factors to buffer against life’s stresses*” [17].This model has been used in young people to improve their general health [18, 19], as well as their sexual and reproductive health [20–22]. Nonetheless, the assets that influence searching for a response to SV have not been addressed in depth in studies on sexual violence, but rather in studies on dating violence [23].

Some studies have identified formal SV support facilitators such as the presence of empathic staff, the perception of comfort and safety and maintaining confidentiality [9]. Furthermore, certain services or support facilities are necessary in order to address assaults that have occurred and publicize available resources [12, 24]. The use of new technologies has been recognized as a factor that effectively promotes awareness about SV and formal support resources [25]. In terms of more informal supports, young people’s relatives and friends are considered an important source of support [10, 26].The asset model adds value to this positive perspective of identifying facilitators because it emphasizes “the positive capability to identify problems and activate solutions” [17].

Most studies in the field of SV on factors that influence support-seeking include young people’s perceptions, [9, 10, 12, 24, 26] but few analyze the perspectives of professionals [25]. Due to the importance of a positive approach for the development of prevention and care strategies, the objective of this study was to identify the main assets that positively influence searching for formal SV support among young people. This study uses the health assets framework to identify the positive factors that contribute to young people’s search for support for situations of SV. Furthermore, it identifies the different functions of the assets and the level of proximity as perceived by young people and professionals. The results of the study are intended to contribute to the improvement of strategies to deal with sexual violence in the young population.

## Methods

### Sample scope and design

Qualitative study conducted in Spain between September 2020 and October 2021. A total of 38 interviews were carried out with 23 young people between 18 and 24 years of age and 15 professionals who work with young people at services and/or offer specialized care for SV. The identification and selection of professionals and the recruitment strategy was carried out using the snowball method. This consisted of an initial online prospecting of potential participants, the online dissemination of informative materials on the study and contacting the networks of identified entities. The research team developed criteria for the selection of participants based on the objectives of the study. The choice of Spain as the context for the study points to the need to take into account the structural and regional variability of socio-economic resources and policies in the field of sexual violence. Therefore, the sample of young people was heterogeneously designed in terms of age, sex, level of education, place of origin and geographical distribution, including different regions in the country: Madrid, Catalonia, the Basque Country, Region of Valencia, Navarre and the Balearic Islands. The total population of these regions represents around 50% of the total Spanish population in 2021. The criteria selection for the professionals related to the type of support center where they were employed, the scope of action and the geographical distribution in the aforementioned regions. Table 1 shows the characteristics of the participants. Young men and women were not recruited because they were victims of sexual violence. However, during the interviews, some young women reported that they had been exposed to SV, or that they knew others (women and men) who had also experienced it. None of the young men identified themselves as perpetrators of SV. We considered important the engagement of both young women and young men in this study, due to the likelihood of SV experiences among them. The sample was completed when discursive saturation was reached (See Table 1).

**Table 1:** Profile of interviewees, young people and professionals

**Interviews with young people**					**Interviews with professionals**			
**Code**^**a**^	**Age**	**Sex**	**Education**	**Origin**	**Code**^**a**^	**Sex**	**Type of service**	**Management**
**W_01**	23	Woman	University studies	National	**VVCS_31**	Woman	Assistance service for SV victims	Public Administration
**M_02**	24	Man	Non-university studies	Migrant	**VVCS_32**	Woman	Assistance service for SV victims	Public Administration
**M_03**	23	Man	Non-university studies	National	**VVCS_34**	Woman	Association specializing in SV victims assistance	Civil Society Organization
**M_04**	19	Man	University studies	National	**VVCS_37**	Woman	Assistance service for GBV victims	Public Administration
**M_05**	18	Man	University studies	National	**VVCS_38**	Woman	Feminist association specializing in GBV victims assistance	Civil Society Organization
**W_06**	19	Woman	University studies	National				
**M_07**	18	Man	University studies	National	**VVCS_39**	Woman	Assistance service for GBV victims	Public Administration
**M_08**	19	Man	University studies	National				
**M_09**	19	Man	University studies	National	**VVCS_44**	Woman	Association specializing in SV victims assistance	Civil Society Organization
**W_10**	23	Woman	University studies	National				
**W_11**	20	Woman	University studies	National	**SY_33**	Woman	Social theater and education association with SV awareness programs	Civil Society Organization
**W_12**	22	Woman	Non-university studies	Migrant				
**W_13**	19	Woman	Non-university studies	Migrant	**SY_35**	Man	LGTBI+ association specialized in raising awareness about sexual diversity	Civil Society Organization
**M_14**	24	Man	Non-university studies	Migrant				
**M_15**	23	Man	Non-university studies	National	**SY_36**	Man	Association for intervention and social inclusion with awareness programs about SV	Civil Society Organization
**W_16**	23	Woman	Non-university studies	Migrant				
**W_17**	23	Woman	Non-university studies	Migrant	**SY_40**	Woman	Educational and Vocational Training Center	Public Administration
**M_18**	24	Man	University studies	National				
**W_19**	19	Woman	Non-university studies	National	**SY_41**	Man	Sociocultural and leisure association	Civil Society Organization
**M_20**	25	Man	Non-university studies	Migrant	**SY_42**	Woman	Sports Club	Civil Society Organization
**M_21**	18	Man	Non-university studies	National	**SY_43**	Woman	Municipal youth council	Public Administration
**W_22**	19	Woman	Non-university studies	National	**SY_45**	Woman	University	Public Administration
**W_23**	24	Woman	Non-university studies	Migrant				

Source: Author’s elaboration

*SV* Sexual violence, *GBV* Gender-based violence

^a^The code indicates the person’s gender if they are young (*W* Women, *M* Men) or type of resource for professionals (*VVCS* Victims of Violence Care Services, *SY* Services for Youth)

### Data collection

Thirty-eight semi-structured interviews were conducted lasting between 50 and 70 min. Due to the COVID-19 pandemic, the interviews were held by telephone (26) and video call (12). The interview format was chosen by the interviewee. The interviews were recorded. Verbal and non-verbal communication strategies were developed to minimize the barriers related to distance. Interviewer and interviewee were grouped according to sex for the interviews with young people. The aim was to offer a space of trust, where intimate and sensitive details could be discussed. Interviews were conducted by two interviewers, a man and a woman, with experience in qualitative research methodologies and gender-based violence.

Two open thematic scripts (See Table 2) were used and adapted to each profile (young people and professionals) that were tested and reviewed in the initial phases of the field work. Specifically, they were tested during the first interviews of three young people and three professionals.

**Table 2:** Final structure of the interview scripts

**Guide for young people**	1) Knowledge of existing resources to address SV.
	2) Difficulties and facilitators in accessing and using support resources.
	3) Assessment of support resources in terms of availability, information, adaptability and assessment of informal resources.
**Guide for professionals**	1) Description and assessment of the resource center where they work.
	2) Difficulties and facilitators of access and use of the support resource by young people.
	3) Assessment of the resources (positive aspects, possible improvement, recommendations on accessibility and use).

Source: Author's elaboration

### Analysis

An inductive and theoretical thematic analysis [27] was carried out by coding the interview transcriptions with the support of Atlas.ti 9 software. Firstly, a pre-analysis of the entire content of the interviews was carried out. This analysis was based on a coding tree that responded to the objectives of the study and included the following: knowledge of support resources; mechanisms of access to services; barriers to access, facilitators and use of support resources; prevention; and approach. The interviews were double coded, and the information was triangulated between two analysts. Following an inductive method, codes with similar content were grouped and classified into potential themes. These were discussed and negotiated by the analysts. This content was divided into two blocks 1) Professionals and other referents’ responses to SV in young people; and 2) Assessment of the response by the participants.

Following this first analysis, a more theoretical thematic analysis was carried out, adapting the constant comparative method, under the grounded theory approach [28], to interpret the inferred data from the conceptual framework of health assets. The dataset analysed was the one related to block 1 obtained from the pre-analysis. It should be noted that the “asset” category is based on the researchers’ interpretation of responses. The interviews did not include the word “assets”, rather they referred to resources that support responses to SV. In other words, the operational definition of assets was established, and with this the facilitating resources that fit the concept were sought. Once assets were identified, a more in-depth analysis was conducted that followed inductive thematic analysis, with two main themes: 1) Comparison of the identified assets and the functions assigned to them by young men and women; and 2) Comparison of the perceptions of the response to SV between young people and professionals.

Within the first main theme related to the assets identified by young people, an additional exhaustive analysis was conducted to categorize the identified assets. The assets identified by young people were categorized along two axes:

1. An axis of “formality”, where the most formal assets correspond to those that are most institutionalized (judicial or police system), and the most informal assets correspond to elements of a person’s surroundings (friends, family, teachers). The most formal assets are those in which rules and routines persist over time and have rigid regulation (police, health system…). In this way the organization is less dependent on the individuals that make it up. In contrast, the less formal resources (family or friends) are those in which rules, routines and members are less defined. Therefore, they depend more on individuals. This categorization was prepared by the analysts.
2. An axis related to the level of perceived proximity or accessibility of the assets to the young people. This categorization aligns with young women’s perceptions observed in the interviews.

Also, an additional categorization was carried out to facilitate interpretation. The assets were located, as well as the functions perceived by young people, in a sequence of phases of the SV response process: from earlier phases of prevention, such as promoting healthy affective sexual relationships, to later phases of response, such as provision of care for SV victims.

Finally, the second theme of analysis was carried out by comparing the perceptions of professionals and young people about the relationship between the assets identified and their functions.

This article was adapted according to the methodological recommendations of SRQR [29] and COREQ [30] (See Tables 1 and 2 in Additional file 1). On the other hand, the quotes from the participants were translated from Spanish into English by a professional translator at the time of writing the article.

### Ethical approval

Participation in interviews was voluntary and unpaid. Informed consent was obtained from all participants both verbally and by recording. Approval was obtained by the Ethics Committee of the University of Alicante (UA-2020-07-07). In addition, some ethical considerations were taken to minimise the impact of the interviews on a sensitive topic such as sexual violence among young people. As mentioned above, in the case of young people, gender was taken into account in the choice of interviewer. On the other hand, the preferences and rhythms of the participants were taken into account during the fieldwork. Also, in cases where unaddressed cases of SV were detected during the interviews, it was suggested to the young people to discuss these situations with a specialist.

## Results

The results are shown below, structured by the main themes of analysis. The first theme of analysis, titled “*Comparison of the identified assets and the functions assigned to them by young men and women*”, is presented in several subsections. The results of the second theme of analysis, titled “*Comparison of young people’s and professionals’ perceptions of the support responses to SV*” describe differences between the two groups in their identification of assets.

### Comparison of identified assets and the functions assigned to them by young men and women

#### Assets identified by the young interviewees

The assets identified by young women and men were similar (See Table 3). They both indicated informal assets, such as groups of friends, family and couples, to which they related functions typical of an initial response to SV (See Table 4). They assigned each asset actions, such as raising awareness, for which the next M_05 quote serves as an illustrative example, or not leaving a potential victim alone, accompanying them home or calling them during their journey. They are also recognized as assets with an auxiliary role in the initial phases of victimization, such as providing the necessary emotional support to continue seeking professional help, as can be seen from the following W_06 quote.

M_05: “Thanks to many friends of mine, who are activists, I’ve been informed a lot about sexual violence, gender violence […] now I believe I have enough information to know how to deal with each situation, and correct people if they say inappropriate things”

W_06: “I’ve spoken to the girl and I’ve encouraged her to report it and my friends told her they can be witnesses, they were there and know what happened”

**Table 3:** Assets Identified by Young People and Professionals

	**Young people**		**Professionals**
	♀	♂	
	Friends	Friends	Friends
	Partner	Partner	-
	Family	Family	Family
	Key informant women	-	-
	Feminist associations and initiatives	-	-
	Internet and social networks	Internet and social networks	Internet and social networks
	Cultural and advertising products	Cultural and advertising products	Cultural and advertising products
	Traditional media	Traditional media	-
	-	Associations and social resources for integration, youth	Associations and social resources for integration, youth
	Specialized GBV and SV helplines	Specialized GBV and SV helplines	Specialized GBV and SV helplines
	Specialized GBV and SV services	Specialized GBV and SV services	Specialized GBV and SV services
	Education system	Education system	Education system
	Healthcare system Including psychological care	Healthcare system Including psychological care	Healthcare system Including psychological care
	-	Criminal police-judicial system	Criminal police-judicial system

Source: Author’s elaboration

**Table 4:** Functions that young people attributed to assets for SV response

		**Assets**											
		**Close surroundings**		**Feminist assets**		**Social communication spaces**		**Entities working with youth**		**Specialized GBV and VS services**		**Other institutions**	
**Functions**		**Friends + partner**	**Family**	**Key informant women**	**Feminist associations and initiatives**	**Internet and social networks**	**Cultural and advertising products + Traditional media**	**Social resources for integration, youth…**	**Education system**	**Specialized GBV and SV helplines**	**Specialized GBV and SV services**	**Healthcare system**	**Criminal police-judicial system**
**Promoting healthy sex-affective relation-ships**	**Creating healthy spaces for socialization**	√	√		√	√	√	√	√				
	**Safe space to share experiences**	√	√		√	√		√	√			√	
**Informing**	**Conceptualization of SV**	√	√		√	√	√	√	√			√	
	**Awareness raising**	√			√	√	√	√	√		√		
	**Specific training in gender and violence**				√				√				
**Preventing direct violence**		√	√	√									√
**Violence detecting**		√	√	√								√	
**Approaching violence immidiately to minimize it**		√		√					√		√		
**Channeling and disseminating other assets**		√	√	√	√	√	√	√	√	√	√	√	√
**Holding the attacker acount-able**	**Publicly highlighting cases of SV**	√			√	√							√
	**Re-educating the attacker**	√										√	
**Suppporting the victim**	**Emotional support**	√	√	√	√	√		√				√	
	**Economic and material support**		√								√		
	**Professional therapy and legal support**										√	√	√

Source: Own elaboration

At an intermediate level of formality, the interviewees refer to a type of asset that analysts have called social communication spaces, as they determine public opinion and socially shared conceptions of SV. This includes the traditional media (TV, press), as well as the internet and social networks, and cultural, artistic and advertising products. The young people assigned the function of offering examples for healthy sex-affective dynamics to these assets, as well as acting as a channel for information and to disseminate support resources. However, they perceive that the internet and social networks transcend these functions. In this regard, young people are not passive subjects, as they can interact, share their personal SV experiences, as the next W_10 quote mentions, and even publicly highlight cases of SV, by seeking to hold the perpetrator accountable.

W_10: “Well mainly from the television, all media, quite a lot as well on social media. I do read a lot of news on social networks, even testimonies from girls […]. On social networks, I do think it’s broader and there are testimonies from different girls and different news that people upload that aren’t on the television. I do think maybe a bit more visibility is given to sexual violence, than television as such. I still think it’s a bit taboo, in my opinion”

Both the young men and young women interviewees recognize formal assets, such as the health system and psychological services. The fact that they almost exclusively attribute to the latter the function of re-educating the attacker stands out. Furthermore, they identified specialized resources for addressing GBV, whose main action is to provide professional support for victims of SV, including their material or financial support, as it is transcended from the following W_17 quote. Meanwhile, GBV and SV helplines were designated as a specific asset, justified by their key function of referring victims to specific SV and GBV support resources. Another asset identified was the education system, as a healthy space for socialization, awareness (as the next W_01 quote shows) and specific training on gender-related violence, as well as a space to learn about other assets, such as feminist associations, GBV-specialized resources, helplines, police, etc.

W_01: “When I took a course on equality at university […] when I broke up with him […] in class they explained gender violence to me […]. I started to realize and I was like, oh wow, this isn’t right! And so, I told my friends more”

W_17: “There’s a center…for women where if you’ve suffered any kind of sexual violence or male violence, you can go and there are two psychologists, I think, I think they have two […]. They also refer you to an assistant who helps you with things. I don’t know the procedure well to be honest, but anyway, something like that”

Ultimately, there are two factors that are perceived by both sexes as catalysts for the proper functioning of the assets. On the one hand, the participants value that in contexts where there is a high degree of associationism, the presence and familiarity of these entities among young people increases, therefore improving their capacity for community impact. The next W_19 quote illustrates this perception.

W_19: “My town is well organized, so I also understand that if I go to other towns the same doesn’t happen, right? But I do think there’s starting to be more things, more awareness […] Now I see loads of guys who are undoing things […] trying to change or at least listen”

On the other hand, some young people believe that asset effectiveness is mediated by individual willingness. This is expressed in attitudes such as becoming educated on the issue out of one’s own interest, as the W_06 quote illustrates, or by directly responding to a SV situation experienced by oneself with a positive and critical coping attitude, as the next W_01 quote shows.

W_01: “I have a lot of feminist self-defence…that’s what the workshop is called. It doesn’t mean standing up to a guy but getting out of there and running. I think we now have a lot of tools […] Saying: “Look, bro, if you continue to send me these photos, one, I’ll publish them on Instagram”. […] “I haven’t asked you for anything”. […] I’ve seen a bit of this direct action by girls”

W_06: “Because I’m really interested in the topic, well I’ve spoken to different people about it and…I’ve come up in my mind what I think, what’s right, after speaking to a lot of people and debating, finding information. […] I’ve done it out of interest, just like maybe I ask my sister, or you ask her the same and she doesn’t know how to answer because she has no idea on the topic. And we’ve been to the same place and been with the same teachers”

#### Differences among young women and men in identifying assets

The most notable difference between young men and women in this study is related to the lack of identification, by men, of so-called “feminist” assets: women key informants and feminist groups and initiatives. Key informant women are personal contacts that are not part of the immediate surroundings yet are perceived as a useful and reliable resource if someone experiences SV, either because of their knowledge on gender and support resources or because of the assurance or empowerment they project. They are assigned functions of detection and immediate action to respond to violence when it is taking place, whether that may come in the form of helping the victim, or confronting the attacker, as the following W_17 quote implies, or disseminating resources to respond to SV and facilitate referral to other assets, as it is illustrated by W_19 quote.

W_17: “One night I was out having some beers, […] the girl comes up to me…and says to me: “Hey, could you take me home? I don’t want him to go with me” […] And she explained to me what had happened in the club. And I said […] Let’s go, ok? […] I’ll walk you home and then I’ll come back […]”

W_19: “Well, I, because personally, I know people who are more involved, so I’d go directly to them, right? […] I personally would call them, say hey, I want information on this, I want to be informed on this”

The interviewed young women reported having found a safe space in feminist associations (self-organized groups, meetings…), and in cultural or artistic feminist initiatives, whether of an individual or group nature. In addition to raising awareness and dissemination, the interviewees assigned the function of carrying out more specific gender equality training to these associations, as it is transcended from W_06 quote. They also relate them to functions focused on more advanced support responses to SV, including carrying out direct prevention, detecting violence as it occurs and immediately addressing it to stop or minimize it.

W_06: “I’m in a feminist group […] we send each other news […] We’re informed, we find articles, we look for books about the topic, we might look for a video that a girl has uploaded to social networks […] each person shares their opinion or tells us if something similar has happened to them and how it could be avoided”

In terms of the male interviewees, some identified other kinds of social and neighborhood associations and support resources as an asset, as well as those dedicated to youth and integration, which were not identified by the young women. These resources can range from more formal institutions, dependent on social services, to leisure associations for young people, although the type of function attributed to them are similar. This is why they were included as a single asset. Similarly, to what feminist associations represent for young women, they find a safe space to share their sex-affective experiences, obtain information, seek emotional support or request help and guidance if they experience SV themselves, as the following M_02 quote shows.

M_02: “Social educators, so, in the neighborhood there are lots of centers […]. If I have a problem like this, normally I send them a message and they say: “well go here...and ask”, normally or “Wait, I’ll look for the phone number and you can call”

In contrast to the young women, the young men identified the judicial and police system as an asset, regardless of whether the person who is sexually assaulted is a man or a woman. They believe it is urgent to report SV in order to hold the attacker accountable and to prevent violence towards other victims, as the M_20 quote illustrates. Some young men do experience SV, but they do not identify GBV resources as assets, as they believe such resources are not intended for them, thus they only support legal solutions, as M_03 quote seems to imply.

M_20: “The first measures are to report it, seek help and immediately report if sexual violence has occurred […] as soon as possible, so the girls can prove with evidence in the hospitals […] and, therefore, a legal process begins against the accused, the rapist*”*

M_03: “If I were someone who suffered sexual violence as a woman, I’d call 016 and they would know to put me in contact with an association or something. As I am a man, what would I do? Well, the police, that’s it, I don’t have anything else…that’s it.”

In contrast, despite considering the police and judicial system an institutional stakeholder that takes part in SV response, the young women did not see it an asset, source of help or facilitator, but rather as a barrier. They indicated that victims of SV suffer from revictimization and that the culture of masculinity within the police force generates distrust, as the next W_19 quote illustrates. In addition, they considered that not all types of SV could be addressed within the legal framework, such as digital SV mentioned in the following W_01 quote.

W_01: “You can go to the police station and say, “look what he’s sending me, what do I do?” I think that…well, they ignore you a bit and say something like “well, he’ll get over it”[...]”

W_19: “Go and report it and they ask you […] “And why did you go to his house or why did you leave the club with him…?” […] and question everything you do to challenge your testimony. […] “So, why am I going to report it if they aren’t going to go anything and they question me during…I don’t know how many hours, it’s worse”

The young men see themselves as an asset, as friends or family members that meet the function of preventing or addressing immediate violence, protecting the victim (as in the next M_21 quote) and reprimanding the attacker (as in the following M_02 quote).

M_21: “My mum, if she needs to go anywhere strange, I’m the first person to go with her. […] I’d be really hurt if something like that would happen to someone like that, someone close”.

M_02: “I don’t know, I don’t know what the reaction would be by those around me, but my reaction would be to say a few things. […] Like “Hey, stop, look what you’re doing”[...]”

However, young women were also perceived as assets in functions such as educating and raising awareness among their friends, partners and family, as both following quotes by W_17 and W_12 illustrate.

W_17: “In the beginning my partner wasn’t interested in any of this […] But after 2 or 3 months of talking to him and saying “darling, this is important”, he started to see it, and now […] he’s one of the few men able to listen and say wow, this is crazy”

W_12: “With my brother […] who is 16, as a man, I try to say things like “hey, that’s not right”. […] I try, as a woman, to give him a perspective, especially to the men in my family and surroundings, of what I wouldn’t like to be done to me, so they don’t do it […] to other women”

#### Perceptions of proximity to assets and their level of formality

**Fig. 1:**
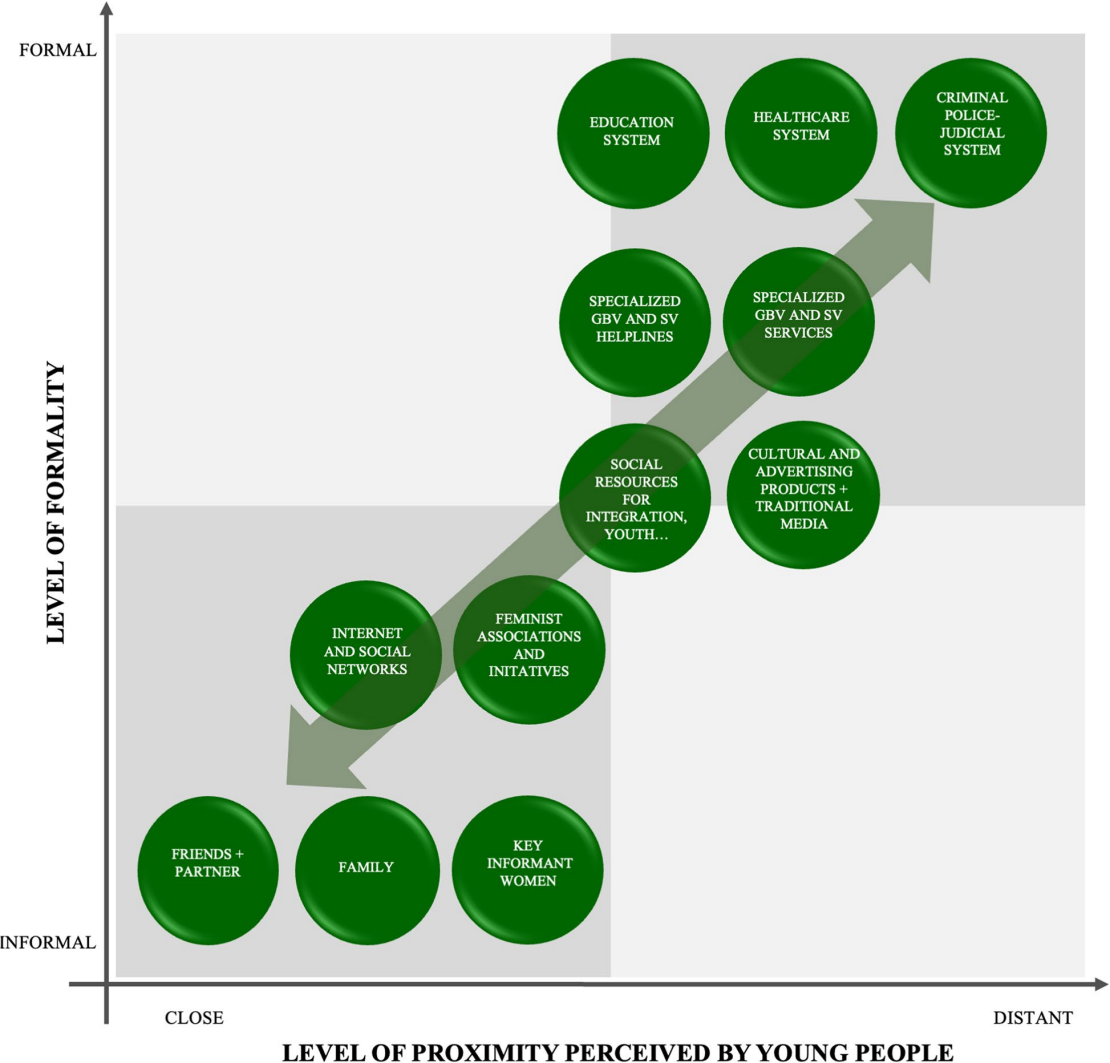
Symbolic representation of assets on the axes of formality/proximity perceived by young people. Source: Own elaboration

In this study, the level of formality of the different assets had a negative relationship with the proximity as perceived by young people (See Fig. 1). Interviewees understood proximity in terms of accessibility and familiarity, in addition to physical and geographic proximity, as M_03 quote shows. Young people experience help provided by formal support resources as less close, less personal and less accessible in terms of geographic proximity than informal resources.

M_03: “I think that it’s proximity […] For any type of tool to work, whether it’s the police or an association […], for it to be really effective […] it needs to be at the level, well of town halls, so the minimum unit of the administration […] the results are better”

Young people also discussed proximity in a symbolic sense, which is related to the adaptability of assets to youth: knowledge of their realities and experiences with SV, mechanisms of access to services (as in W_10 quote), their language and even their conceptualization of SV (as in M_04 quote).

W_10: “That first approach to that service […] for it to not be in person […] would make it easier, because the person, who has to go to that place, and is seen going in and so […] maybe by telephone or if information can be given on social networks, WhatsApp and so […] or videocall”

M_04: “[GBV resources] are much more focused on what’s within a sex-affective relationship itself, of people maybe a bit older, more mature […] Outside a sex-affective relationship and even the younger population, maybe there’s a little bit missing”

In general, informal assets that are part of one’s immediate and daily surroundings are considered to be more accessible and closer. Among these, they highlighted that when talking about SV they prefer to talk to friends or their partner rather than family, when the attack has occurred outside their relationship. In comparison with their partner and friends, they reported that family is more unapproachable and distant, because they are afraid of not being understood, as the next M_07 quote implies.

M_07: “I think young people usually speak to our closest environment […] or partner before talking to our family”

At an intermediate level of formality, young people reported that the traditional media also generate a certain distrust and a sense of distance. However, young men and women did perceive social networks and the internet as common and accessible spaces. They felt similarly in terms of more informal organizations, such as feminist groups and neighborhood and youth associations, that seem more connected to their experiences as young people, as it is transcended by the next W_11 quote. In fact, they suggested that this type of “grassroots” organization could act as a bridge between people who need SV support and the more formal institutions that are in charge of addressing SV.

W_11: “Groups that so to say are really out there, right? They’re not full of bureaucracy, in the end, or not influenced by an institution or interests. […] They can work on the problem […] from a place of trust […] I think that institutions have forgotten that these groups exist, that there’s an entire grassroots social movement”

The higher formality assets, especially the criminal police and judicial systems and the healthcare system, were perceived as distant and difficult to access when searching for SV. In addition, young people associated the high level of formality of care resources and specialized GBV helplines with failing to adapt to the realities of young people, as M_03 quote illustrates. The education system was an exception among formal SV resources. In general, they perceived it as closer in proximity.

M_03: “They’re too institutional. I see them as too archaic; they’re ultimately bureaucratic. In other words, I see them as very little human, very little connected to social reality. […] I don’t think it’s a question of there being no tools, but rather whether these tools are really capable of providing a solution for these young people”

### Perceptions of SV support responses: a comparison of the perspectives of young people and professionals

**Fig. 2:**
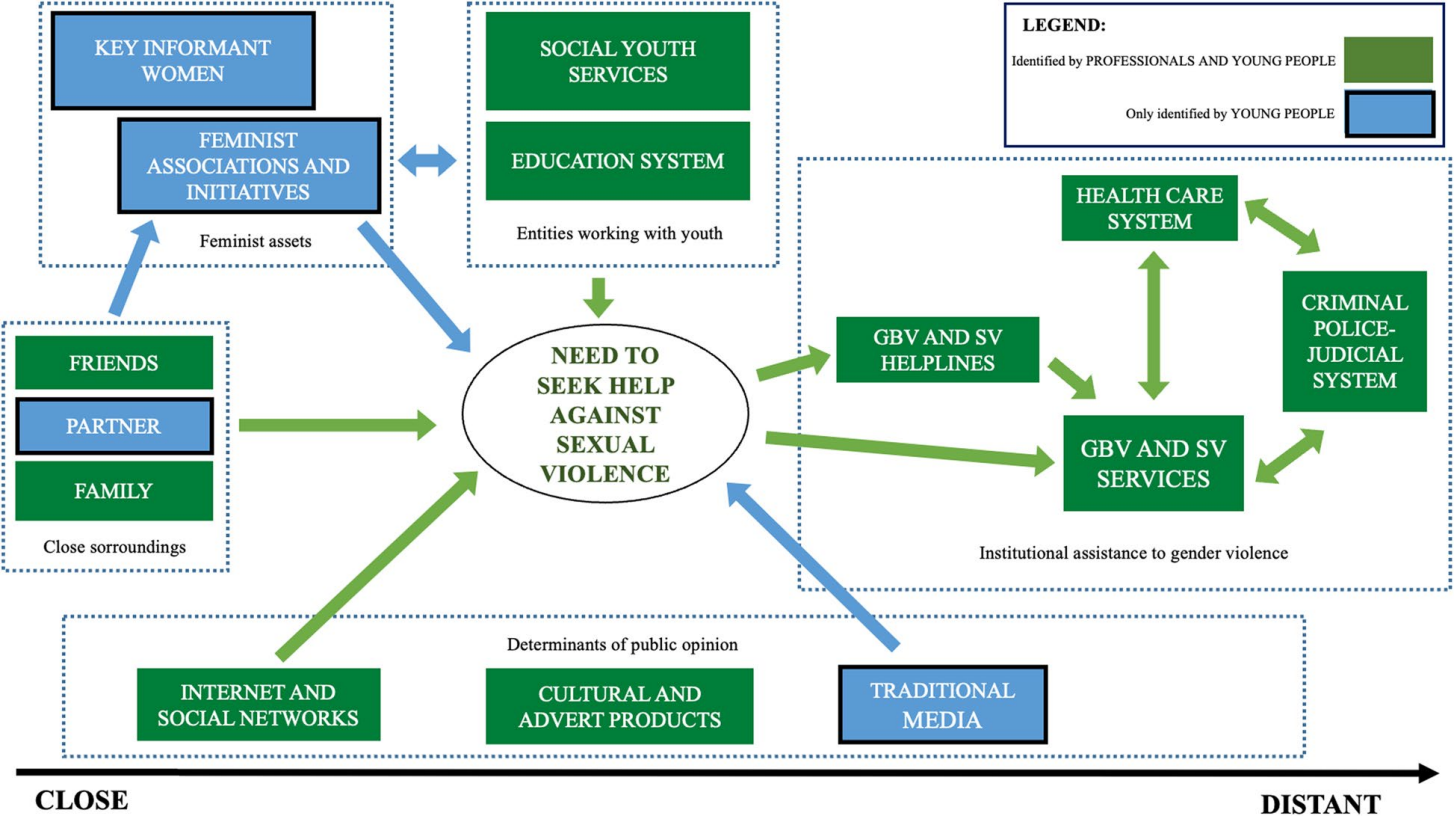
Flow diagram of relationships between assets for SV response. Source: Own elaboration

The professionals interviewed did not mention any SV asset that was not also identified by the young men and women interviewed in this study (See Table 3 and Fig. 2). However, young people identified feminist assets, which was a resource not identified by the professionals. The young women indicated that feminist assets play a key role in their own responses to SV. The professionals also failed to identify partners or the traditional media as assets in SV support.

In terms of the functions of each support resource or asset, professionals identified three types beyond the 14 functions identified by young people (See Table 4). They were assigned the titles: “research in SV and GBV”, “establishment of action protocols for SV and GBV”, as VVCS_38 quote suggests, and “network coordination with other entities”, as shown in SY_43 quote. The first two functions are carried out primarily by services specialized in GBV, while the third is carried out by both those addressing GBV and those that work with young people (education centers, associations, etc.)

VVCS_38: “We have drafted documents […] theoretical frameworks on violence, more in a general sense […] for the government […] and sexual violence and feminist protocols”

SY_43: “We’re going to do a joint consultancy with the Equality [council] […] we need to look for that support network with colleagues to work”

Regarding relationships between assets (See Fig. 2), both groups of study participants seemed to perceive a flow of information and specific cases from a low level of formality, in the initial phases of the response process, towards the specialized approach offered by institutionalized SV response assets. Feminist assets are perceived as of close proximity to the victims and to determinants of public opinion. These assets help by intervening in the detection of SV and supporting the victims’ search for support among specialized services, even those that involve a lack of trust due to their high level of formality. According to the perspectives of study participants, referral is made through SV and GBV helplines or by directly going to care resources or the health system.

The comparison of the perspectives of young people and professionals shows a referral channel that is a priority for young women but is not clearly recognized by professionals: referral through feminist assets. The professionals do not identify this option, which, according to the perceptions of the young women, involves friends and the education system. Information and referral flows go from friendships to feminist associations and initiatives (See Fig. 2). Furthermore, there is a bilateral flow between these associations and the education system. Many feminist associations emerge within this system (student associations), and they influence the system by bringing in new discourses and exerting pressure to modify practices. This process is illustrated by the following W_19 quote.

W_19: “For example, a student came to a feminist group at high school, to complain that a classmate had started touching her breasts and bottom in class […] and at least try to help and that’s it. […] so, they can get it off their chest […] we’ve tried to tell them, well, ask for help and so. Some have and others haven’t and so we’ve continued to listen to them […] making it clear that we aren’t psychologists and, in the end, real help is somewhere else”

## Discussion

This study shows similarities and discrepancies among young women and men and SV response professionals in identifying assets for seeking SV support among youth. The study identifies functions attributed by young people to each type of support and shows an inverse relationship between perceptions of proximity and the level of formality of the asset (less formal assets such as family and friends are perceived as more accessible). This study also contributes to map of relationships and information exchanges between assets. The main findings of this study include the identification of feminist assets as essential for the young women, not recognized by young men or by professionals, and the fact that women do not identify institutional resources for SV support as assets, such as the police and judicial system.

The informal assets for SV support identified by the participants, such as family and friends coincide with some of the facilitators found in other studies [10, 17, 18, 22, 23]. However, in these studies, partners are not identified as factor to improve young people’s ability to seek SV support. The family is considered an asset because it acts as a protective factor against dating violence in young people [31]. Likewise, it was observed that support from friends and peer groups improves the willingness of young people to help in a situation of SV. This has been identified in other studies, as friends serve as support networks due to their great influence on young people’s attitudes and behaviours [20, 32, 33].

Young women identify themselves as assets as they raise awareness and create a change in SV response. However, young men identify more with intervening in cases of violence, in other words, protecting the victim, as well as “scolding” the attacker. These functions are essential in generating a trend in young people’s surroundings so that others will act in the same way [32]. These self-perceptions are related to traditional gender roles that are built upon patriarchy: women as caregivers and transmitters of knowledge and men as protectors. It is interesting to observe that, although young people are becoming increasingly aware of the structure of inequality in gender, they also have roles that reproduce that same structure. Organizing educational interventions that consider approaches to eliminating social constructions of gender is currently a societal issue [34–36].

Some of the young people in this study identified their partners as an asset to offer emotional support in SV situations. Furthermore, from a health promotion perspective, programmes aimed at partners could be beneficial in reinforcing positive behaviours among youth [22]. The fact that young people can talk about reproductive and sexual health with their sexual partners is a protective factor against SV within the relationship, as this fosters equal gender attitudes [37]. In SV outside an intimate relationship, this also encourages couples to improve their emotional support, increasing a victim’s coping capacity.

In terms of formal assets, the education system was identified by both groups of participants. It is the only formal resource that was perceived as of close proximity for young people. Support from teachers enables equal awareness among young men and women [23], therefore helping young people to identify and face normalized SV situations, such as sexual harassment, even online. In the case of SV and GBV, support from teachers can help young people to perceive education centers as a safe space to seek support [38]. Programmes have been carried out in the educational and social spheres that focus on raising awareness about dating violence, myths, power, traditional gender roles and resources available to victims and attackers [39, 40]. This kind of intervention has resulted in significant changes in young people, in terms of both physical and psychological violence, such as SV [41–43]. Thus, education interventions have been developed to promote healthy relationships among youth by promoting assets to solve situations of violence in relationships [21, 23, 44]. This allows young people to value the assets they have in their surroundings and ask for support and improve their coping capacity [23]. These interventions have also been effective in reducing benevolent sexism among the young population [45, 46] and violent experiences in their affective sexual relationships [21]. It would be interesting to approach address SV in the fields of reproductive and sexual health. When a SV situation is observed, this would allow these organizations to identify themselves as assets and improve the response to SV by demystifying the myths that justify sexual assault [47].

For young people, informal assets are closer in proximity when seeking SV support. On the other hand, the police and health systems, with a high level of formality, are perceived as distant. It is interesting to consider the social construction of gender that young people have of the police and judicial systems. Young men identify the legal route as a direct path to put an end to the situation. However, they believe it to be a distant asset, most likely due to the social perception of the police which is usually negative, because of stereotyped beliefs about police officers [48]. Additionally, this negative perception is related to the mistreatment of citizens, thus influencing the legitimacy of the police [49, 50]. Thus there is a need to adapt resources to young people, as they do not seem youth-friendly enough [51]. Meanwhile, young women identify this kind of asset as a barrier due to re-victimization and lack of trust generated by the culture of masculinity of the police force. This barrier has been identified in other studies and may be related to the lack of training in GBV and SV within the police force [13, 14, 52, 53].

Regarding referral channels, young people and the professionals perceive a flow that extends from assets with a low level of formality (family, friends, the Internet or cultural products) towards more specialized assets (institutional resources). Due to the relevance of the victim’s social network and after identifying the referral relationships between assets, it is necessary to work with the community to promote healthy affective sexual relationships and raise awareness to improve prevention, detection and accompaniment for the victim [24]. Thus, society and the victim’s environment are encouraged to be aware of the available resources. Training potential witnesses of SV to intervene effectively and safely to prevent and act on SV is important [54–57].

### Limitations and strengths

Regarding the limitations of this study, most young people tend to refer to severe SV situations to identify support assets. On the other hand, intentional sampling is subject to selection bias for two reasons: 1) because interviewees might be more understanding of SV, and 2) because migrants might be less likely to be university students. These results may be applicable to other contexts with which our results have been compared. Nonetheless, in order to improve transferability, a detailed description of the study sample has been drafted. Regarding the strengths, credibility was guaranteed with double coding and triangulation of the information. A critical review of the results ensured that they are reliable. Finally, in order to guarantee reliability, an inductive analysis approach was used, as well as a detailed description of the methodological process and literal quotes [58].

### Study implication for research and policy

Most of the interviewed professionals in this study did not recognize as assets partners, feminist associations and key informants. These feminist assets have been identified as key collaborating agents for the incorporation of the gender perspective in community health. This approach takes into account the health assets model and the formative functions of feminist groups are similar to those identified in our study [59]. Along these lines, within the asset’s framework, women’s activist groups have been observed to act as agents that help increase resilience to threats in poor communities [60]. In the context of sexual violence, the feminist assets highlighted in our study have not been clearly identified in the literature. That is, assets as a fundamental gear for the process of seeking support in the face of SV. However, peer support within groups of survivors of SV [61] has been identified as an asset. These types of groups, may be organized within feminist associations, helping to increase the resilience of individuals and mitigate the negative effects of SV [61–65] For these reasons, it is necessary to value these assets so professionals can actively work with them and mitigate the gap that exists between these collectives and institutions. Future research should aim to explore the reasons why professionals do not identify these assets and how they can be incorporated into the support services’ response. This would help in the daily practice of professionals. It would also be advisable to continue research on health assets as it provides new evidence that is not identified through the analysis of barriers alone [16]. Finally, it would be advisable to sensitise and train the identified assets so that they know how to provide the appropriate support and information when faced with a situation of SV.

## Conclusions

The results of this study aim to improve SV care for young people, from a health asset perspective and taking into consideration both the youth and professionals’ perspectives. Both have identified factors that improve SV victims’ help-seeking capacity. They also coincide in most assets. However, professionals and young men did not identify feminist assets that are key factors for young women, as they have greater prevalence of SV. Once the referral channels are analyzed, it is necessary to work with the community by raising awareness to promote accompanying victims through a formal network of resources. This awareness raising can be carried out by feminist groups or through educational interventions that enable both young men and women to understand the problem of SV, to provide adequate support to victims and to know where to go for help when faced with SV. For this reason, it is important for professionals to be aware of the assets that they learn about from victims of violence against women, and for there to be communication with them. To this end, it is important to inform professionals about the assets that professionals do not acknowledge, and about actions that allow young people to access formal resources.

### Supplementary Information

Additional file 1: Complementary information on the methodology of the study. Table 1. Standards for Reporting Qualitative Research (SRQR). Table 2. Consolidated criteria for reporting qualitative studies (COREQ): 32-item checklist (COREQ).

## Data Availability

The datasets used and/or analysed during the current study are available from the corresponding author on reasonable request. Information about study participants will not be made available.

## Abbreviations

SV: Sexual violence
GBV: Gender-based violence
W: Women
M: Men
VVCS: Victims of Violence Care Services
SY: Services for Youth
