# Supplementary material for: “Thanks to my activists Friends”: a qualitative study of perspectives of young adults and professionals on the factors related to seeking support among victims of sexual violence in Spain
Source: Arch Public Health. 2024 Jun 21;82:93. doi: 10.1186/s13690-024-01319-z (PMC11191312; doi:10.1186/s13690-024-01319-z)
Supplement: Supplementary file 1 — Additional file 1: Complementary information on the methodology of the study. Table 1. Standards for Reporting Qualitative Research (SRQR). Table 2. Consolidated criteria for reporting qualitative studies (COREQ): 32-item checklist (COREQ). [file 13690_2024_1319_MOESM1_ESM.docx]

**Supplementary Information**

**Complementary information on the methodology of the study.**

**Table 1. Standards for Reporting Qualitative Research (SRQR)**

| **Topic** | **Item information** | **No. page** |
| --- | --- | --- |
| **Title and abstract** | | |
| **Title** | Concise description of the nature and topic of the study Identifying the study as qualitative or indicating the approach (e.g., ethnography, grounded theory) or data collection methods (e.g., interview, focus group) is recommended | 1 |
| **Abstract** | Summary of key elements of the study using the abstract format of the intended publication; typically includes background, purpose, methods, results, and conclusions | 1 |
| **Introduction** | | |
| **Problem formulation** | Description and significance of the problem/phenomenon studied; review of relevant theory and empirical work; problem statement | 3 |
| **Purpose or research question** | Purpose of the study and specific objectives or questions | 3 |
| **Methods** | | |
| **Qualitative approach and research paradigm** | Qualitative approach (e.g., ethnography, grounded theory, case study, phenomenology, narrative research) and guiding theory if appropriate; identifying the research paradigm (e.g., postpositivist, constructivist/ interpretivist) is also recommended; rationaleb | ✔  **Methods> Analysis** |
| **Researcher characteristics and reflexivity** | Researchers’ characteristics that may influence the research, including personal attributes, qualifications/experience, relationship with participants, assumptions, and/or presuppositions; potential or actual interaction between researchers’ characteristics and the research questions, approach, methods, results, and/or transferability | ✔  **Methods> Data collection >**  Grouping interviewee-interviewer according to gender |
| **Context** | Setting/site and salient contextual factors; rationaleb | ✔ |
| **Sampling strategy** | How and why research participants, documents, or events were selected; criteria for deciding when no further sampling was necessary (e.g., sampling saturation); rationaleb | ✔  **Method > Sample scope and design** |
| **Ethical issues pertaining to human subjects** | Documentation of approval by an appropriate ethics review board and participant consent, or explanation for lack thereof; other confidentiality and data security issues. | ✔  **Method > Ethical considerations** |
| **Data collection methods** | Types of data collected; details of data collection procedures including (as appropriate) start and stop dates of data collection and analysis, iterative process, triangulation of sources/methods, and modification of procedures in response to evolving study findings; rationaleb | ✔  **Method > Data collection** |
| **Data collection instruments and technologies** | Description of instruments (e.g., interview guides, questionnaires) and devices (e.g., audio recorders) used for data collection; if/how the instrument(s) changed over the course of the study | ✔  **Method > Data collection** |
| **Units of study** | Number and relevant characteristics of participants, documents, or events included in the study; level of participation (could be reported in results) | ✔  **Method > Data collection** |
| **Data processing** | Methods for processing data prior to and during analysis, including transcription, data entry, data management and security, verification of data integrity, data coding, and anonymization/deidentification of excerpts | ✔  **Method > Data collection** |
| **Data analysis** | Process by which inferences, themes, etc., were identified and developed, including the researchers involved in data analysis; usually references a specific paradigm or approach; rationale b | ✔  **Methods> Analysis** |
| **Techniques to enhance trustworthiness** | Techniques to enhance trustworthiness and credibility of data analysis (e.g., member checking, audit trail, triangulation); rationaleb | ✔ |
| **Results/findings** | | |
| **Synthesis and interpretation** | Main findings (e.g., interpretations, inferences, and themes); might include development of a theory or model, or integration with prior research or theory | ✔ |
| **Links to empirical data** | Evidence (e.g., quotes, field notes, text excerpts, photographs) to substantiate analytic findings | ✔ |
| **Discussion** | | |
| **Integration with prior work, implications, transferability, and contribution(s) to the field** | Short summary of main findings; explanation of how findings and conclusions connect to, support, elaborate on, or challenge conclusions of earlier scholarship; discussion of scope of application/ generalizability; identification of unique contribution(s) to scholarship in a discipline or field | ✔ |
| **Limitations** | Trustworthiness and limitations of findings | ✔ |
| **Other** | | |
| **Conflicts of interest** | Potential sources of influence or perceived influence on study conduct and conclusions; how these were managed | ✔ |
| **Funding** | Sources of funding and other support; role of funders in data collection, interpretation, and reporting | ✔ |

**Table 2. Consolidated criteria for reporting qualitative studies (COREQ): 32-item checklist. (COREQ)**

| No. Item | Guide questions/description | Reported |
| --- | --- | --- |
| Domain 1: Research team an reﬂexivity | | |
| **1. Inter viewer/facilitator** | Which author/s conducted the  interview? | The authors have experience in qualitative research and are researchers in the field of public health, sociology, nursing and women’s health in all phases of sexual and reproductive life (midwifery). Furthermore, the members of Cooperativa Aplica have training and experience in qualitative analysis and qualitative research methodologies. |
| **2. Credentials** | What were the researcher’s credentials? |  |
| **3. Occupation** | What was their occupation at the time of the study? |  |
| **4. Gender** | Was the researcher male or female? | The interviews with the young people were conducted by two women with the girls and one man with the boys. The interviews with the professionals did not have this difference. |
| **5. Experience and training** | What experience or training did the researcher have? | The interviewers had sufficient training and experience in qualitative research methodology. |
| **6. Relationship with participants established** | Was a relationship established prior to study commencement? | No |
| **7. Participant knowledge**  **of the interviewer** | What did the participants know about  the researcher? | The participants were informed when they were contacted and at the beginning of the interview on the project, scope and goals, the research leader and the university responsible for it. |
| **8. Interviewer**  **characteristics** | What characteristics were reported  about the inter viewer/facilitator? |  |
| Domain 2: study design | | |
| 9. Methodological  orientation and Theory | What methodological orientation was  stated to underpin the study? | Content analysis + interpretive and comparative analysis according to gender and young people vs. professionals. |
| 10. Sampling | How were participants selected? | The identification, selection and recruitment strategy of participants was carried out by the snowball technique. On the one hand, the researchers did so online with an initial survey of potential participants and disseminating information on the study. On the other hand, they did so through a network of contacts of the identified entities. Afterwards, selection was intentional by differentiating the profiles and their selection criteria. |
| 11. Method of approach | How were participants approached? |  |
| 12. Sample size | How many participants were in the study? | 38 participants (23 young people and 15 professionals) |
| 13. Non-­‐participation | How many people refused to participate or dropped out? Reasons? | There was no loss of participants due to purposive sampling. |
| 14. Setting of data collection | Where was the data collected? | 26 interviews were carried out by telephone and 12 by video call. |
| 15. Presence of non-­‐  participants | Was anyone else present besides the  participants and researchers? | No |
| 16. Description of sample | What are the important characteristics of the sample? | A series of criteria to select the participants were considered. On the one hand, the following variables were taken into account for the young people: 1) age (18-21 years and 22-24 years to reach a balanced representation); 2) sex; 3) level of education (university or non-university); 4) place of origin (migrant or national); 5) geographical distribution according to region. On the other hand, in order to select the resource, the following factors were considered: 1) type of resource management (public administration or third sector entities); 2) scope of action (address SV and youth services); 3) geographical distribution according to region. |
| 17. Interview guide | Were questions, prompts, guides provided by the authors? | The interview script was drafted by the members of the research team and was adapted to each profile. The scripts were tested and adapted in the initial phases of the field work. |
| 18. Repeat interviews | Were repeat interviews carried out? | No |
| 19. Audio/visual recording | Did the research use audio or visual recording to collect the data? | Audio recording was used to collect data from telephone interviews. |
| 20. Field notes | Were ﬁeld notes made during and/or after the interview? | The interviewers were instructed to take notes immediately after the interview, focusing on issues of the situation and on rapport and connection between interviewers and interviewees. Interview notes added context and were verified to see if discursive saturation had been achieved, but they were not used in the analysis. |
| 21. Duration | What was the duration of the interviews? | The duration of the interviews was 50 to 70 minutes. |
| 22. Data saturation | Was data saturation discussed? | Yes. The saturation of the discourse occurred when the last interviews did not generate additional information and it was decided to finalize the selection of participants. |
| 23. Transcripts returned | Were transcripts returned to  participants for comment and/or correction? | No |
| Domain 3: analysis and ﬁndings | | |
| 24. Number of data coders | How many data coders coded the data? | Two analysts of the research team. |
| 25. Description of the  coding tree | Did authors provide a description of the  coding tree? | Yes, the codes included in the coding tree that was made for the first part of the content analysis are listed. |
| 26. Derivation of themes | Were themes identiﬁed in advance or derived from the data? | The codes used to analyze the transcriptions were based on the project objectives and interviews. They were used for a pre-analysis. Based on that, new data codes emerged. From these new codes, categories and identified topics emerged. |
| 27. Software | What software, if applicable, was used to manage the data? | *Atlas.ti (version 9)* |
| 28. Participant checking | Did participants provide feedback on the ﬁndings? | No |
| 29. Quotations presented | Were participant quotations presented to illustrate the themes/ﬁndings? Was  each quotation identiﬁed? | The most representative quotes of the discourses found are added to the manuscript in the results section. |
| 30. Data and ﬁndings consistent | Was there consistency between the data presented and the ﬁndings? | *Yes* |
| 31. Clarity of major themes | Were major themes clearly presented in the ﬁndings? | Yes |
| 32. Clarity of minor  themes | Is there a description of diverse cases or discussion of minor themes? | Yes |
